# Foodborne Outbreaks Caused by Human Norovirus GII.P17-GII.17–Contaminated Nori, Japan, 2017

**DOI:** 10.3201/eid2405.171733

**Published:** 2018-05

**Authors:** Naomi Sakon, Kenji Sadamasu, Takayuki Shinkai, Yousuke Hamajima, Hideaki Yoshitomi, Yuki Matsushima, Rika Takada, Fumio Terasoma, Asako Nakamura, Jun Komano, Koo Nagasawa, Hideaki Shimizu, Kazuhiko Katayama, Hirokazu Kimura

**Affiliations:** Osaka Institute of Public Health, Osaka, Japan (N. Sakon, R. Takada);; Tokyo Metropolitan Institute of Public Health, Tokyo, Japan (K. Sadamasu, T. Shinkai);; Wakayama Prefectural Research Center of Environment and Public Health, Wakayama, Japan (Y. Hamajima, F. Terasoma);; Fukuoka Institute of Health and Environmental Sciences, Dazaifu, Japan (H. Yoshitomi, A. Nakamura);; Kawasaki City Institute for Public Health, Kawasaki City, Japan (Y. Matsushima, H. Shimizu);; National Hospital Organization Nagoya Medical Center, Nagoya, Japan (J. Komano);; Chiba University, Chiba, Japan (K. Nagasawa);; Kitasato University, Tokyo (K. Katayama);; Gunma Paz University, Takasaki, Japan (H. Kimura);; National Institute of Infectious Diseases, Tokyo (H. Kimura)

**Keywords:** norovirus, GII.P17-GII.17, dried shredded seaweed, nori, school lunch, food handler, high infectivity, foodborne disease, outbreaks, viruses, Japan, food safety

## Abstract

Seven foodborne norovirus outbreaks attributable to the GII.P17-GII.17 strain were reported across Japan in 2017, causing illness in a total of 2,094 persons. Nori (dried shredded seaweed) was implicated in all outbreaks and tested positive for norovirus. Our data highlight the stability of norovirus in dehydrated food products.

Seven foodborne norovirus outbreaks were reported in 4 remote areas across Japan during January–February 2017, causing illness in 2,094 persons (Figure). In all outbreaks, norovirus GII.P17-GII.17 was detected in stool specimens of patients. Food survey results indicated that dried shredded seaweed (nori) was served before all outbreaks. Norovirus was also detected in nori-containing dishes. An investigation revealed that the nori served in each instance was manufactured by the same food processing company.

**Figure:**
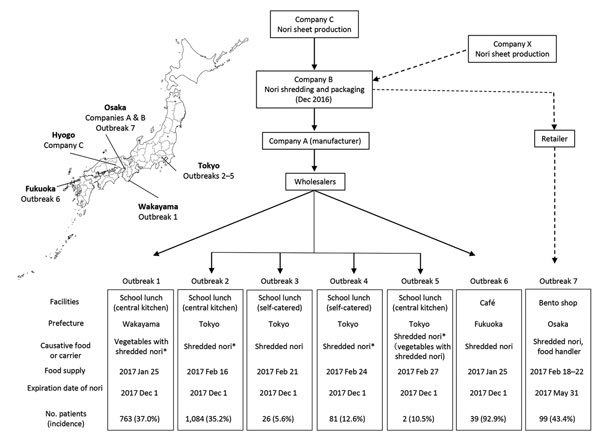
Overview of foodborne norovirus outbreaks associated with dried shredded nori during the 2016–17 endemic season, Japan. Production and distribution of dried, shredded nori products and the 7 norovirus outbreaks are outlined. Solid and dashed lines indicate the production and distribution of 2 distinct lots. The asterisk indicates detection of norovirus in food, shredded nori, or both. Inset map shows geographic locations of outbreaks and manufacturers.

## The Study

Outbreak 1 was reported on January 26 in 4 kindergartens, 6 elementary schools, and 5 junior high schools in the prefecture of Wakayama, Japan (*1*). A total of 1,943 children and 119 school staff members had eaten lunch the day before; of these, 678 (34.9%) children and 85 (71.4%) school staff members had acute gastroenteritis. Norovirus was also detected in the stool specimens from 10 of the 27 food handlers in the central kitchen who had eaten the same lunch. Food survey results indicated that boiled vegetables mixed with shredded nori was responsible for this outbreak.

Outbreak 2 was reported on February 17 in 7 elementary schools in the city of Tachikawa, part of metropolitan Tokyo, Japan (*1*). A total of 1,084 (35.2%) of the 3,078 persons who ate the lunch served the day before had gastrointestinal symptoms. The lunch included shredded nori as a topping on cooked rice.

Outbreaks 3 and 4 were reported in separate, self-catered school lunch settings on February 22 and 25 in the city of Kodaira, also part of metropolitan Tokyo (*1*). The numbers of patients with gastrointestinal symptoms were 26 (5.6% attack rate) in outbreak 3 and 81 (12.6% attack rate) in outbreak 4. Shredded nori was served as a topping on cooked rice in both outbreaks.

Outbreak 5 was reported in Western Tama, also in metropolitan Tokyo, on February 27. A central kitchen served school lunch to 19 persons, and 2 of them (10.5%) had gastrointestinal symptoms. Shredded nori was served with boiled vegetables.

Outbreak 6 was reported on March 9. After conducting retrospective surveillance, the Kurume city government in Fukuoka prefecture announced that a foodborne outbreak occurred in a business office on January 25 that was attributable to a nori product with the same expiration date as the nori implicated in outbreaks 1–5. A total of 39 (92%) of the 42 employees who had eaten at the office’s café had gastrointestinal symptoms. Shredded nori had been served as a salad topping.

Outbreak 7, announced by the Osaka prefectural government, caused illness in 99 persons during February 18–24, including 4 food handlers who consumed a bento box (a single-portion take-out or home-packed meal common in Japanese cuisine). A bento shop provided 228 meal boxes during this period that contained nori product from company B. Those who had eaten from bento boxes without nori also had gastrointestinal symptoms.

We detected norovirus in nori-containing food in outbreak 1 by using the PANSORBIN-trap method (*2*). Tokyo Metropolitan Institute of Public Health tested norovirus from food from outbreaks 2–5 by using the A3T method, by which nori was incubated with the *Proteus vulgaris* NBRC3045 strain (*3*). Among 21 nori samples, including those from the merchandise in stock of the wholesaler and those that remained in the kitchen from outbreaks 2–5 in Tokyo, 7 samples were positive for norovirus GII.17 by nested reverse transcription PCR (*4*–*6*). These data, along with the food survey results, strongly suggest that nori was responsible for the foodborne norovirus outbreaks.

The amount of shredded nori served as a topping on cooked rice was 0.5–1.0 g per dish. The shredded nori contained 360–2,900 copies/g of norovirus genome, a measure that did not take into account the recovery rate of the virus particles from the food (*4*). In outbreak 7, food handlers shared and reused plastic gloves, which increased the risk for norovirus contamination across food.

Nori sheets were originally produced by company C, and food processing company B shredded them to 2-mm width and packed them with desiccants (Figure). Approximately 800 packages were produced, shipped to company A on December 10 and 27, 2016, and sold under the company A brand with the same expiration date. Another batch was produced by company B in December 2016 and sold under its own brand with an expiration date of May 31, 2017. These packages were stored at an ambient temperature.

On February 27, 2017, the Osaka city government announced that norovirus GII.17 was detected from 8 of 25 environmental wipe samples at company B. These samples were subjected to ultracentrifugation to precipitate norovirus particles before nucleic acid extraction. Samples collected from the nori sheet shredding equipment, a telephone handle, and the toilet were positive for norovirus. At company B, an employee who was responsible for the shredding step had gastrointestinal symptoms before the production of the nori in question in late December 2016 but nevertheless continued working. No outbreak had been reported after the products were recalled, and the business of company B was suspended. Investigators suspected that the nori was contaminated with norovirus during the shredding process at company B.

Most foodborne outbreaks in Japan are attributable to norovirus GII.P17-GII.17, a novel variant that emerged in Asia during 2014–2015 (*7*,*8*). The genome sequence spanning the N terminus of the major capsid protein (*VP1*) gene, a short 302-nt region that is routinely sequenced, was identical among the GII.P17-GII.17 strains isolated in Japan during the 2016–17 season. Therefore, we sequenced and analyzed 2 virus genes, RNA-dependent RNA polymerase (*RdRp*) (1,530 nt) and *VP1* (1,620 nt). For all 7 outbreaks, the sequence identities of these 2 genes derived from patient stool specimens were 100% identical (Table). As a comparison, we analyzed norovirus GII.P17-GII.17 strains detected from 4 independent outbreaks unrelated to nori products during the same season in Osaka (Table). The sequences of 2 genes among these strains were not identical with those related to nori-associated outbreaks, and phylogenetic tree analyses of the *RdRp* gene showed that sequences associated with the nori-related norovirus outbreaks formed a distinct cluster from the outbreaks unrelated to nori (Technical Appendix Figure). These results and epidemiologic link indicated that all 7 outbreaks were attributable to GII.P17-GII.17–contaminated shredded nori.

**Table:** Genetic analysis of norovirus GII.P17-GII.17 isolates detected in clinical specimens from outbreaks during the 2016–17 endemic season, Japan*

Outbreak or strain	GenBank accession nos.	Isolation date	Identity with outbreak 1 strain, %	
			*RdRp*, 1,530 nt	*VP1*, 1,620 nt
Outbreak 1	LC318755–LC318758	Jan 2017	NA	NA
Outbreak 2	LC258403†	Feb 2017	100	100
Outbreak 6	LC311767–LC311773	Jan 2017	100	100
Outbreak 7	LC318751–LC318754	Feb 2017	100	100
Outbreak A:16–064	LC318745	May 2016	99.6	99.9
Outbreak B:16–299	LC318746	Nov 2016	99.9	99.9
Outbreak C:16–292	LC318750	Nov 2016	99.5	99.6
Outbreak C:16–303	LC318749	Nov 2016	99.7	ND
Outbreak D:16–311	LC318747	Dec 2016	99.9	99.8
Outbreak D:16–315	LC318748	Dec 2016	99.8	99.6
GII.17 Kawasaki308	LC037415	Feb 2015	99.6	99.9

*NA, not applicable; ND, not done. †This isolate was registered as a representative strain of outbreaks 2–5 because all the isolates from outbreaks 2–5 showed 100% sequence identity.

## Conclusions

Our study demonstrated that 7 foodborne norovirus outbreaks in 4 remote regions of Japan were attributable to a shredded nori product that was most likely contaminated during manufacturing. From a technical standpoint, the recovery of norovirus genomic RNA from food is not efficient, so identifying the contaminated food can be difficult; however, the PANSORBIN-trap and A3T methods were shown to be useful for this purpose. Sequence analysis of the *RdRp* and *VP1* regions of the norovirus genome enabled us to distinguish nori-related outbreaks involving norovirus GII.P17-GII.17 from other outbreaks involving the same genotype.

Few dry food–associated foodborne outbreaks with norovirus have been reported previously. In 2014, a large norovirus outbreak involving 1,271 persons was reported in which bread was contaminated with norovirus. The contaminated bread was served at school lunch within 2 days after production (*9*). In contrast, in these nori-related outbreaks, the traceback of implicated nori product revealed that the norovirus infectivity remained for >2 months at ambient temperature under dry conditions. However, the percentage of persons with gastrointestinal symptoms gradually decreased from the date of nori production, suggesting a decline in norovirus infectivity over time under dry conditions.

Technical Appendix Phylogenetic tree analysis of norovirus GII.P17-GII.17 strains associated with dried shredded nori–related and unrelated outbreaks during the 2016–17 endemic season, Japan, 2017.
