# Supplementary material for: Foodborne Outbreaks Caused by Human Norovirus GII.P17-GII.17–Contaminated Nori, Japan, 2017
Source: Emerg Infect Dis. 2018 May;24(5):920–3. doi: 10.3201/eid2405.171733 (PMC5938760; doi:10.3201/eid2405.171733)
Supplement: Technical Appendix — Phylogenetic tree analysis of norovirus GII.P17-GII.17 strains associated with dried shredded nori–related and unrelated outbreaks during the 2016–17 endemic season, Japan, 2017. [file 17-1733-Techapp-s1.pdf]

Article DOI: https://doi.org/10.3201/eid2405.171733

Foodborne Outbreaks Caused by Human Norovirus GII.P17-GII.17–Contaminated Nori, Japan, 2017

Technical Appendix

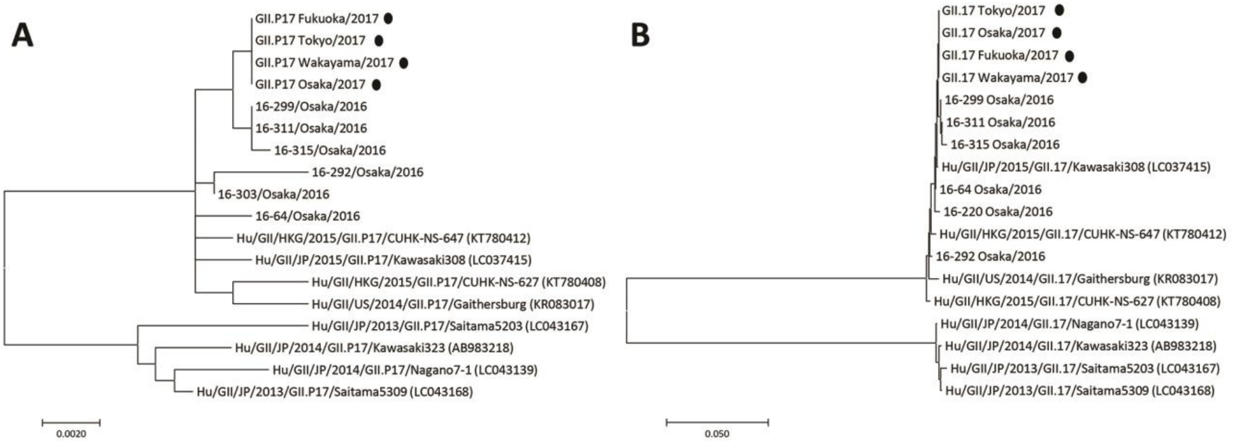

Technical Appendix Figure. Phylogenetic tree analysis of norovirus GII.P17-GII.17 strains associated with dried shredded nori–related and unrelated outbreaks during the 2016–17 endemic season, Japan, 2017. Analyses of viral genes encoding *RdRp* (A) and *VP1* (B) are shown. Solid circles indicate strains from outbreaks with shredded nori. GII.P17 or GII.17_Wakayama/2017 is represented by LC318755, 1 of the 4 strains detected in outbreak 1 (LC318755–LC318758). GII.P17 or GII.17_Tokyo/2017 is represented by LC258403, 1 of the strains detected during outbreaks 2–5. GII.P17 or GII.17_Fukuoka/2017 is represented by LC311767, and GII.P17 or GII.17_Osaka/2017 is represented by LC318751 (Table). The evolutionary analysis was conducted by using MEGA7 software, with the maximum-likelihood method based on the Tamura-Nei model. The phylogenetic tree is drawn to scale, with branch lengths reflecting the number of substitutions per site.
